# Supplementary material for: A Clinico-Genetic Score Incorporating Disease-Free Intervals and Chromosome 8q Copy Numbers: A Novel Prognostic Marker for Recurrence and Survival Following Liver Resection in Patients with Liver Metastases of Uveal Melanoma
Source: Cancers (Basel). 2024 Oct 7;16(19):3407. doi: 10.3390/cancers16193407 (PMC11475758; doi:10.3390/cancers16193407)
Supplement: Supplementary file 1 [file cancers-16-03407-s001.zip › Tables.pdf]

Table S1. Univariate analysis of recurence-free survival (RFS). Cassoux classification was defined as follows: low risk: D3/8nl; intermediate risk: M3/8nl or D3/8g; high risk: M3/8g. TCGA classification was defined as follows: low risk: D3/8nl; intermediate risk: D3/8g; high risk: M3/8g; very high risk: M3/8g with 8q amplification. M3/8nl alterations were classified as intermediate risk. HR: hazard ratio; CI: confidence interval; UM: uveal melanoma; LBD: largest basal diameter; AJCC: American Joint Committee on Cancer. M3: monosomy 3; D3: Disomy 3; 8nl: chromosome 8 normal; 8g: 8q gain

**Variable**	**Univariate analysis of RFS**	
	**HR (95% CI)**	**p-value**
Clinical characteristics		
Age (years), >50 vs ≤50	1.7 (1.1-2.9)	0.03
Gender, Male vs Female	0.9 (0.6-1.5)	0.77
Ocular UM LBD (mm), 10 vs ≤10	0.9 (0.6-1.5)	0.68
T stage AJCC, T4 vs T1/T2/T3	1.4 (0.9-2.3)	0.17
Ciliary body extension, present vs absent	1.8 (1.0-3.0)	0.03
Treatment of ocular UM, enucleation vs iodine disk or protontherapy	1.4 (0.9-2.2)	0.17
Disease-free interval (months), ≤24 vs >24	3.1 (1.9-5.2)	<0.001
Number of liver metastasis on MRI, >2 vs ≤2	1.5 (0.9-2.5)	0.08
Largest liver metastasis on MRI (mm), >20 vs ≤20	1.1 (0.7-1.8)	0.72
Largest liver metastasis on MRI (mm^2), >250 vs ≤250	1.3 (0.8-2.0)	0.29

Capsular miliary disease, present vs absent	1.8 (1.1-2.8)	0.02
Parenchymal miliary disease, present vs absent	1.6 (0.9-3.0)	0.13
Major hepatectomy, absent vs present	0.7 (0.5-1.1)	0.17
Bleeding loss (mL), >100 vs ≤100	1.5 (1.0-2.5)	0.08
Pathological and genetic features		
Liver histopathology, epithelioid or mixed vs fusiform	1.3 (0.8-2.3)	0.34
GNAQ mutation, present vs absent	1.6 (1.0-2.5)	0.07
GNA11 mutation, present vs absent	0.6 (0.4-1.0)	0.06
SF3B1 mutation, present vs absent	0.7 (0.4-1.2)	0.24
BAP1 mutation, present vs absent	1.7 (1.1-2.8)	0.02
EIF1AX mutation, present vs absent	0.7 (0.3-1.7)	0.47
Cassoux classification, intermediate vs high	0.9 (0.5-1.4)	0.58
low vs high	0.5 (0.2-1.3)	0.16
TCGA classification, very high vs high	2.7 (1.5-5.1)	0.001
intermediate vs high	1.5 (0.8-3.0)	0.22
low vs high	0.8 (0.3-2.3)	0.72
8q surgain, absent vs present	0.4 (0.3-0.7)	<0.001

Table S2. Univariate analysis of overall-free survival (OS). Cassoux classification was defined as follows: low risk: D3/8nl; intermediate risk: M3/8nl or D3/8g; high risk: M3/8g. TCGA classification was defined as follows: low risk: D3/8nl; intermediate risk: D3/8g; high risk: M3/8g; very high risk: M3/8g with 8q amplification. M3/8nl alterations were classified as intermediate risk. HR: hazard ratio; CI: confidence interval; UM: uveal melanoma; LBD: largest basal diameter; AJCC: American Joint Committee on Cancer; M3 : monosomy 3; D3 : Disomy 3; 8nl : chromosome 8 normal; 8g : 8q gain

**Variable**	**Univariate analysis of OS**	
	**HR (95% CI)**	**p-value**
Clinical characteristics		
Age (years), >50 vs ≤50	2.0 (1.2-3.5)	0.01
Gender, Male vs Female	0.8 (0.5-1.4)	0.50
Ocular UM LBD (mm), 10 vs ≤10	0.7 (0.4-1.2)	0.21
T stage AJCC, T4 vs T1/T2/T3	1.2 (0.7-2.0)	0.55
Ciliary body extension, present vs absent	1.6 (0.9-2.8)	0.11
Treatment of ocular UM, enucleation vs iodine disk or protontherapy	1.5 (0.9-2.6)	0.11
Disease-free interval (months), ≤24 vs >24	3.6 (2.1-6.2)	<0.001
Number of liver metastasis on MRI, >2 vs ≤2	1.2 (0.7-2.0)	0.52
Largest liver metastasis on MRI (mm), >20 vs ≤20	1.2 (0.7-2.1)	0.57
Largest liver metastasis on MRI (mm^2), >250 vs ≤250	0.9 (0.5-1.5)	0.63
Capsular miliary disease, present vs absent	2.0 (1.2-3.5)	0.009

Parenchymal miliary disease, present vs absent	1.5 (0.8-2.9)	0.21
Major hepatectomy, absent vs present	0.8 (0.5-1.4)	0.44
Bleeding loss (mL), >100 vs ≤100	1.4 (0.8-2.3)	0.21
Surgical liver margins, complete vs incomplete	2.8 (1.2-6.5)	0.02
Pathological and genetic features		
Liver histopathology, epithelioid or mixed vs fusiform	1.6 (0.8-3.0)	0.18
GNAQ mutation, present vs absent	1.3 (0.8-2.3)	0.27
GNA11 mutation, present vs absent	0.7 (0.4-1.3)	0.27
SF3B1 mutation, present vs absent	0.6 (0.3-1.1)	0.08
BAP1 mutation, present vs absent	3.0 (1.6-5.4)	<0.001
EIF1AX mutation, present vs absent	0.4 (0.1-1.3)	0.13
Cassoux classification, intermediate vs high	0.4 (0.2-0.8)	0.008
low vs high	-	>0.99
TCGA classification, very high vs high	2.4 (1.3-4.6)	0.008
intermediate vs high	0.7 (0.3-1.6)	0.38
low vs high	-	>0.99
8q surgain, absent vs present	0.2 (0.1-0.4)	<0.001

Table S3. Multivariable analysis of recurence-free survival (RFS) by genetic classifications. Columns show results from three different multivariable models, each one exploring different genetic classifications (Cassoux classification in model 1; TCGA classification in model 2; 8q surgain in model 3). Multivariable analysis was undertaken entering all variables associated with RFS at the $p < 0.05$ level in univariate analysis, then applying a backward stepwise approach to retain significant factors at the $p < 0.05$ level in the final model. HR: hazard ratio; CI: confidence interval.

Variable	Model 1		Model 2		Model 3	
	HR (95% CI)	p-value	HR (95% CI)	p-value	HR (95% CI)	p-value
Age (years), >50 vs ≤50	-	-	-	-	-	-
Ciliary body extension, present vs absent	-	-	-	-	-	-
Disease-free interval (months), ≤24 vs >24	2.9 (1.7-4.8)	<0.001	-	-	2.2 (1.2-3.8)	0.007
Capsular miliary disease, present vs absent	-	-	-	-	-	-
BAP1 mutation, present vs absent	-	-	2.6 (1.4-4.6)	0.002	-	-
Cassoux classification, intermediate vs high	-	-				
low vs high	-	-				
TCGA classification, very high vs high			2.2 (1.2-4.3)	0.02		
intermediate vs high			1.6 (0.8-3.3)	0.17		
low vs high			0.7 (0.2-2.0)	0.49		
8q surgain, present vs absent					2.2 (1.3-3.7)	0.005

Table S4. Multivariable analysis of overall survival (OS) by genetic classifications. Columns show results from three different multivariable models, each one exploring different genetic classifications (Cassoux classification in model 1; TCGA classification in model 2; 8q surgain in model 3). Multivariable analysis was undertaken entering all variables associated with OS at the $p < 0.05$ level in univariate analysis, then applying a backward stepwise approach to retain significant factors at the $p < 0.05$ level in the final model. HR: hazard ratio; CI: confidence interval

Variable	Model 1		Model 2		Model 3	
	HR (95% CI)	p-value	HR (95% CI)	p-value	HR (95% CI)	p-value
Age (years), >50 vs ≤50	-	-	-	-	-	-
Disease-free interval (months), ≤24 vs >24	3.6 (2.1-6.2)	<0.001	3.6 (2.1-6.2)	<0.001	2.7 (1.5-4.7)	<0.001
Capsular miliary, present vs absent	-	-	-	-	-	-
BAP1 mutation, present vs absent	-	-	-	-	-	-
Cassoux classification, intermediate vs high	-	-				
low vs high	-	-				
TCGA classification, very high vs high			-	-		
intermediate vs high			-	-		
low vs high			-	-		
8q surgain, present vs absent					2.9 (1.6-5.2)	<0.001
