# Supplementary figures and images for: A Clinico-Genetic Score Incorporating Disease-Free Intervals and Chromosome 8q Copy Numbers: A Novel Prognostic Marker for Recurrence and Survival Following Liver Resection in Patients with Liver Metastases of Uveal Melanoma
Source: Cancers (Basel). 2024 Oct 7;16(19):3407. doi: 10.3390/cancers16193407 (PMC11475758; doi:10.3390/cancers16193407)

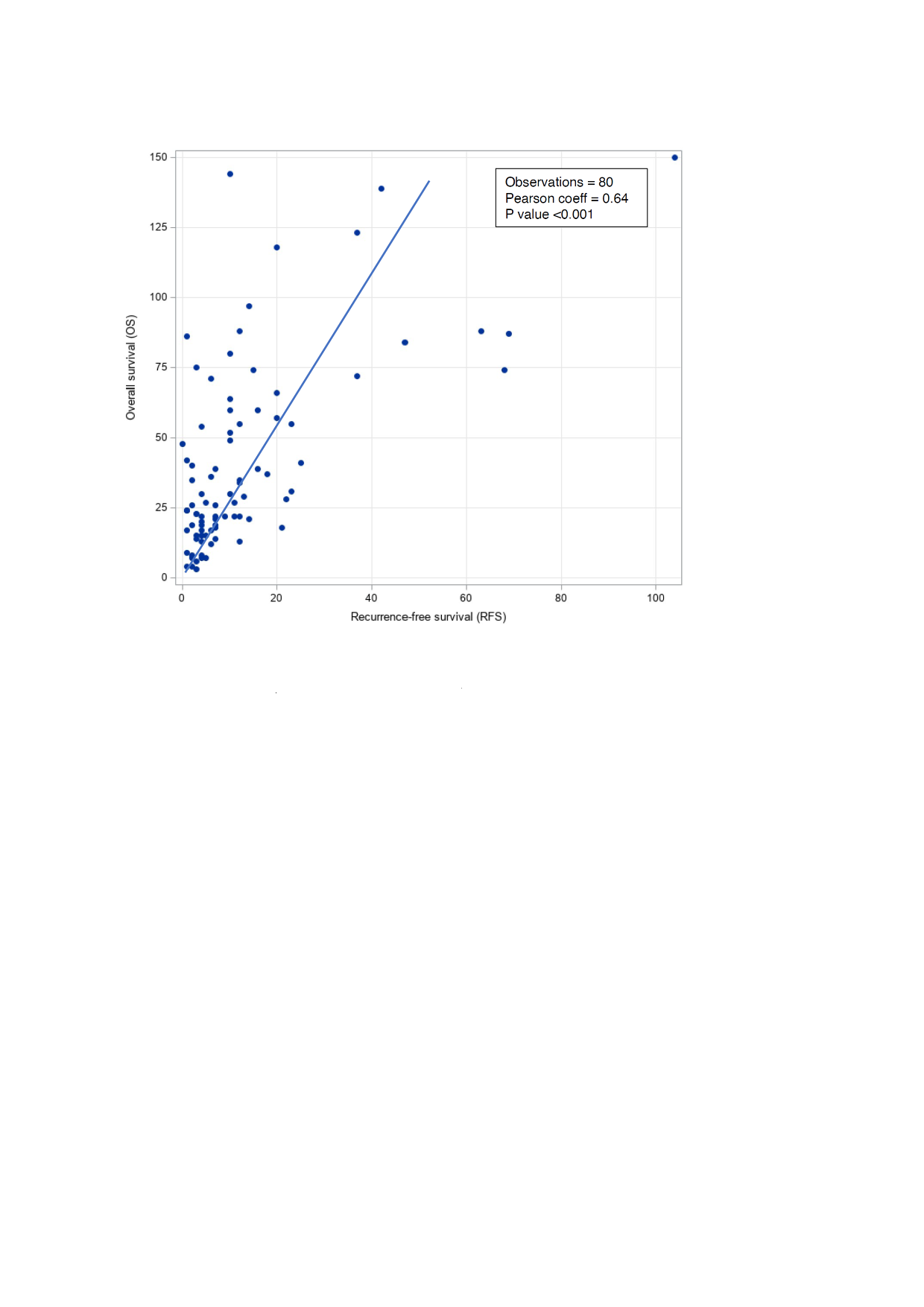

Supplement: Supplementary file 1 [file cancers-16-03407-s001.zip › Figure S1 .tif]

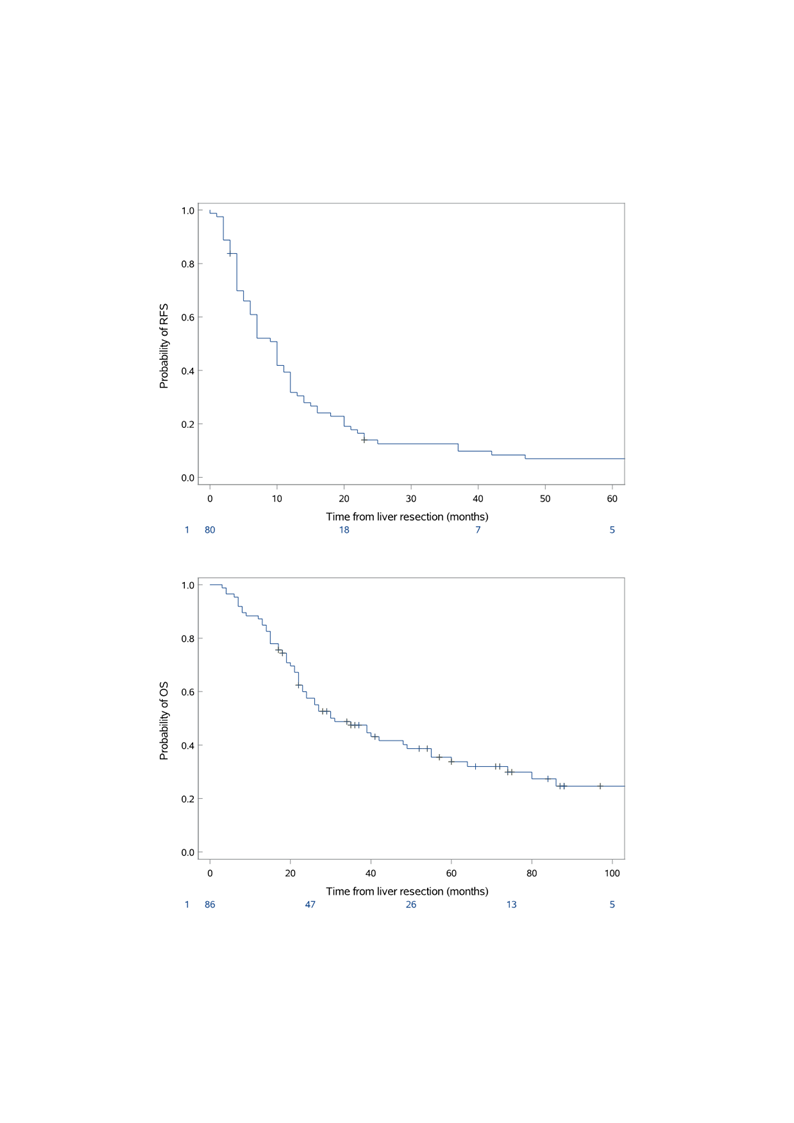

Supplement: Supplementary file 1 [file cancers-16-03407-s001.zip › Figure S2 .tif]

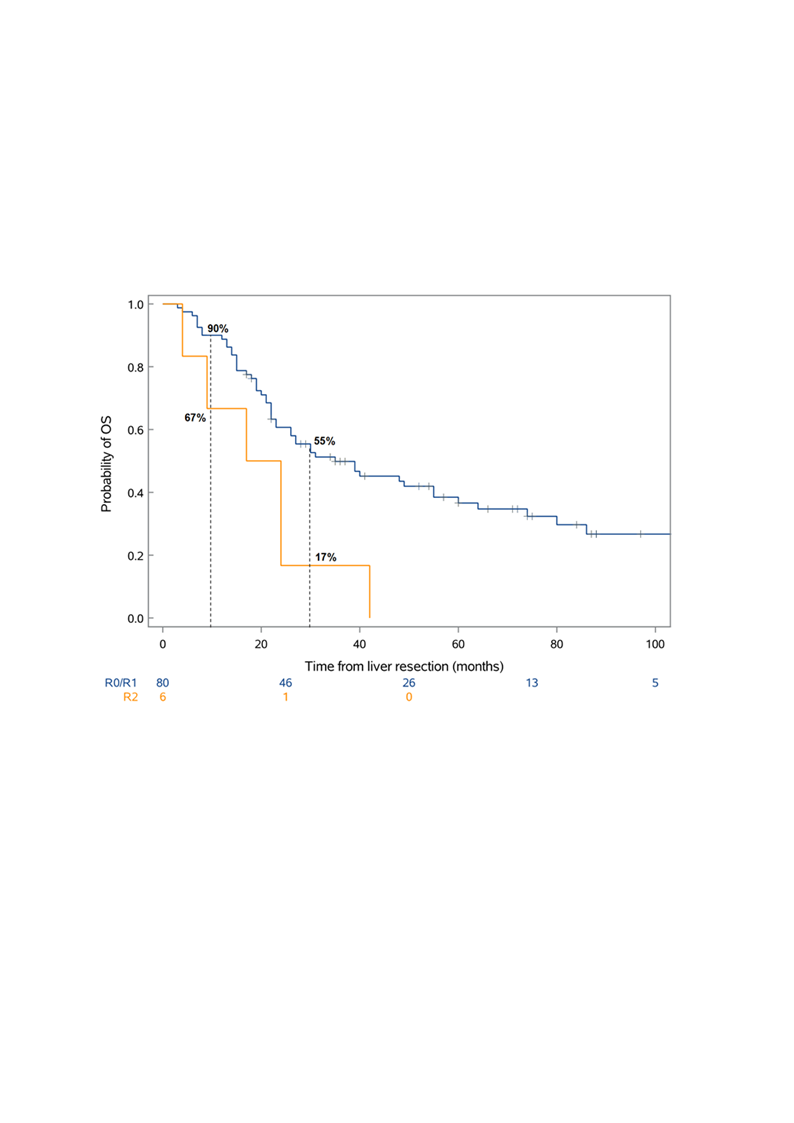

Supplement: Supplementary file 1 [file cancers-16-03407-s001.zip › Figure S3.tif]

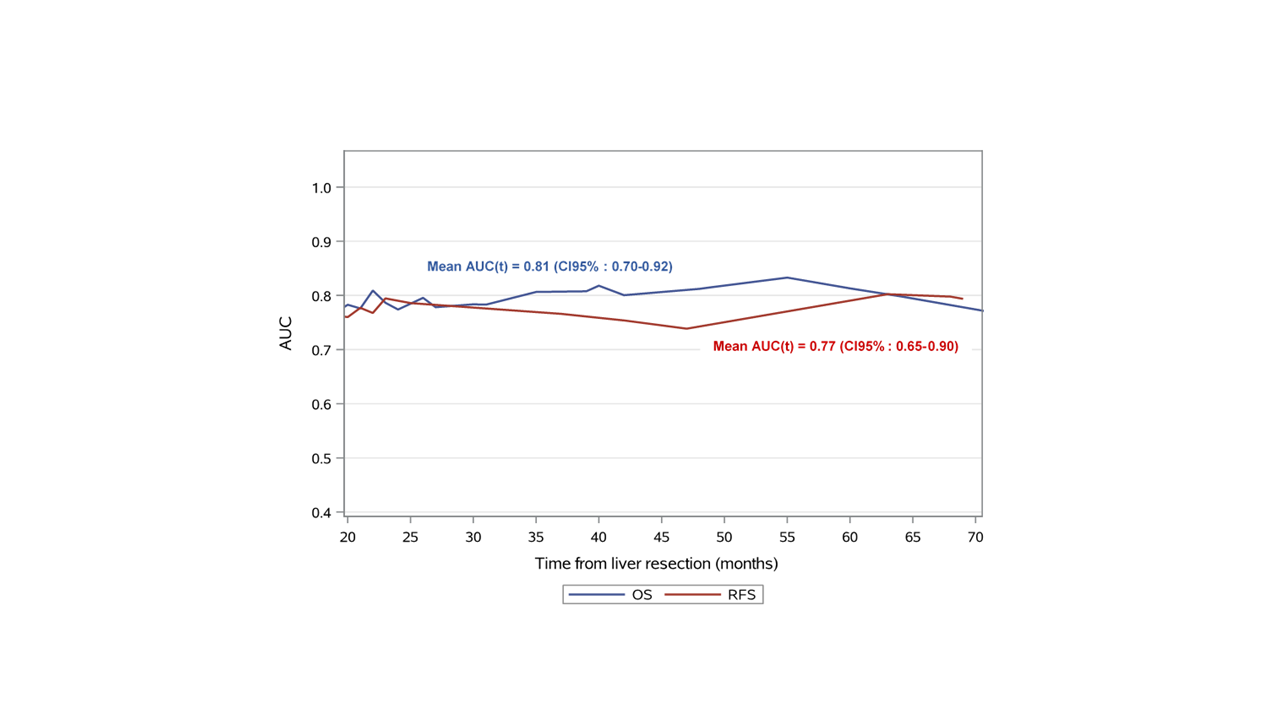

Supplement: Supplementary file 1 [file cancers-16-03407-s001.zip › Figure S4 .tif]

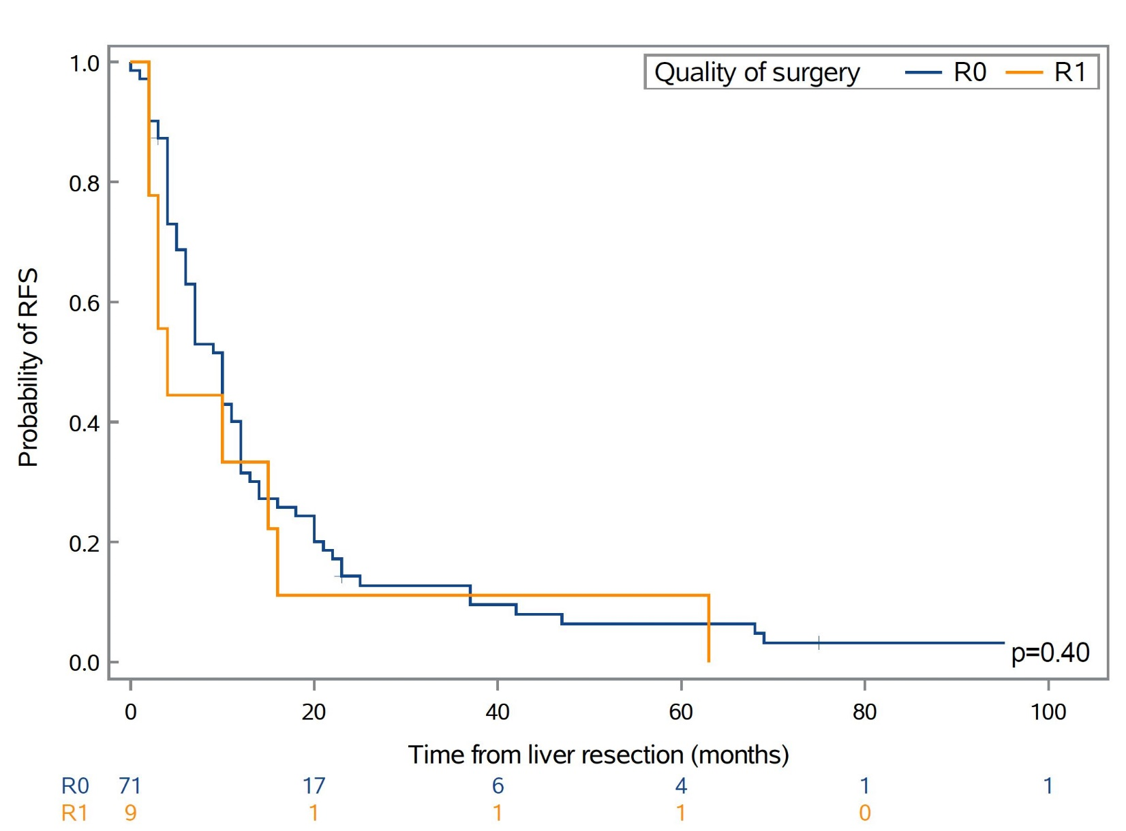

Supplement: Supplementary file 1 [file cancers-16-03407-s001.zip › Figure S5.tif]
